# Author Correction: Coherent coupling between vortex bound states and magnetic impurities in 2D layered superconductors

**DOI:** 10.1038/s41467-025-57925-0

**Published:** 2025-03-13

**Authors:** Sunghun Park, Víctor Barrena, Samuel Mañas-Valero, José J. Baldoví, Antón Fente, Edwin Herrera, Federico Mompeán, Mar García-Hernández, Ángel Rubio, Eugenio Coronado, Isabel Guillamón, Alfredo Levy Yeyati, Hermann Suderow

**Affiliations:** 1 Departamento de Física Teórica de la Materia Condensada, Instituto Nicolás Cabrera and Condensed Matter Physics Center (IFIMAC), Universidad Autónoma de Madrid, Madrid, Spain; 2 Laboratorio de Bajas Temperaturas y Altos Campos Magnéticos, Departamento de Física de la Materia Condensada, Instituto Nicolás Cabrera and Condensed Matter Physics Center (IFIMAC), Unidad Asociada UAM-CSIC, Universidad Autónoma de Madrid, Madrid, Spain; 3 Instituto de Ciencia Molecular (ICMol), Universidad de Valencia, Paterna, Spain; 4 Max Planck Institute for the Structure and Dynamics of Matter, Hamburg, Germany; 5 Instituto de Ciencia de Materiales de Madrid, Consejo Superior de Investigaciones Científicas (ICMM-CSIC), Madrid, Spain; 6 Nano-Bio Spectroscopy Group and European Theoretical Spectroscopy Facility (ETSF), Universidad del País Vasco CFM CSIC-UPV/EHU-MPC & DIPC, San Sebastián, Spain

**Keywords:** Magnetic properties and materials, Superconducting properties and materials

Correction to: *Nature Communications* 10.1038/s41467-021-24531-9, published online 03 August 2021

In the acknowledgements section, the following grant codes were omitted, ‘CEX2019-000919-M and PID2020-114071RB-I00/AEI/10.13059/501100011033)’. The original article has been corrected.

