# Supplementary material for: Coherent coupling between vortex bound states and magnetic impurities in 2D layered superconductors
Source: Nat Commun. 2021 Aug 3;12:4668. doi: 10.1038/s41467-021-24531-9 (PMC8333272; doi:10.1038/s41467-021-24531-9)
Supplement: Supplementary file 1 — Supplementary Information [file 41467_2021_24531_MOESM1_ESM.pdf]

Supplementary Information: Coherent coupling between vortex bound states and magnetic impurities in 2D layered superconductors

Sunghun Park,[1] Víctor Barrena,[2] Samuel Mañas-Valero,[3] José J. Baldoví,[3,4] Antón Fente,[2] Edwin Herrera,[2] Federico Mompeán,[5] Mar García-Hernández,[5] Ángel Rubio,[4,6] Eugenio Coronado,[3] Isabel Guillamón,[2] Alfredo Levy Yeyati,[1] and Hermann Suderow[2]

[1]*Departamento de Física Teórica de la Materia Condensada, Instituto Nicolás Cabrera and Condensed Matter Physics Center (IFIMAC), Universidad Autónoma de Madrid, E-28049 Madrid, Spain*
[2]*Laboratorio de Bajas Temperaturas y Altos Campos Magnéticos, Departamento de Física de la Materia Condensada, Instituto Nicolás Cabrera and Condensed Matter Physics Center (IFIMAC), Unidad Asociada UAM-CSIC, Universidad Autónoma de Madrid, E-28049 Madrid, Spain*
[3]*Instituto de Ciencia Molecular (ICMol), Universidad de Valencia, Catedrático José Beltrán 2, 46980 Paterna, Spain*
[4]*Max Planck Institute for the Structure and Dynamics of Matter, Luruper Chaussee 149, D-22761 Hamburg, Germany*
[5]*Instituto de Ciencia de Materiales de Madrid, Consejo Superior de Investigaciones Científicas (ICMM-CSIC), Sor Juana Inés de la Cruz 3, 28049 Madrid, Spain*
[6]*Nano-Bio Spectroscopy Group and European Theoretical Spectroscopy Facility (ETSF), Universidad del País Vasco CFM CSIC-UPV/EHU-MPC & DIPC, Avenida Tolosa 72, 20018 San Sebastián, Spain*

SUPPLEMENTARY NOTE 1. BOGOLIUBOV DE GENNES CALCULATION OF YSR AND CDGM STATES

Model Hamiltonian for CdGM states. We consider the Bogoliubov de Gennes (BdG) equation describing an isolated vortex at the origin in two dimensions in the basis of electron and hole wavefunctions $\psi = (\psi^+, \psi^-)^T$,

$$\begin{pmatrix} -\frac{\hbar^2\mathbf{k}^2}{2m^*} + E_F & \Delta(\mathbf{r}) \\ \Delta(\mathbf{r})^* & \frac{\hbar^2\mathbf{k}^2}{2m^*} - E_F \end{pmatrix} \psi(\mathbf{r}) = E\psi(\mathbf{r}), \tag{1}$$

where m^* is the absolute value of the effective mass, E_F is the Fermi energy and $\Delta(\mathbf{r}) = \Delta_0 \tanh(r/\xi_V)e^{-i\theta}$ with the size of a vortex core ξ_V. Notice that we consider a system with a hole like band character, as 2H-$NbSe_2$[1]. Otherwise, the diagonal components of the matrix need to be interchanged. As 2H-$NbSe_2$ is a strong type II superconductor with a large penetration depth (of about 200 nm [2, 3]), we can take a constant magnetic field. Spin degeneracy is not included for simplicity, since the wave functions for both spin states are the same. Following previous works [4–6], the CdGM bound state energy and corresponding wave function in the asymptotic region ($r \gg 1/k_F$) in the low energy limit are approximately given by

$$\varepsilon_n = \frac{E_n}{\Delta_0} = (n + \frac{1}{2})\frac{\Delta_0}{2E_F}, \tag{2}$$

$$\psi_n(\mathbf{r}) = \begin{pmatrix} \psi_n^+(\mathbf{r}) \\ \psi_n^-(\mathbf{r}) \end{pmatrix} \tag{3}$$

$$= A_n(r) \begin{pmatrix} e^{-i(n+1)\theta}\cos(k_F r + F_n - \frac{\eta_n}{2}) \\ e^{-in\theta}\cos(k_F r + F_n + \frac{\eta_n}{2}) \end{pmatrix}.$$

Here $A_n(r)$ is

$$A_n(r) = \frac{1}{\sqrt{N_n}} \frac{e^{-\lambda_n(r)}}{\sqrt{k_F \tilde{r}_n}}, \tag{4}$$

where N_n is a normalization factor and $\tilde{r}_n \equiv (r^2 + ((n + 1/2)/k_F)^2)/r$ is introduced to avoid singularities at $r = 0$. n are the eigenstate numbers. λ_n, F_n, and η_n are functions of r given by

$$F_n(r) = \frac{(n+\frac{1}{2})^2 + \frac{1}{4}}{2k_F\tilde{r}_n} - \frac{\pi}{2}\left(\sqrt{\left(n+\frac{1}{2}\right)^2 + \frac{1}{4}} + \frac{1}{2}\right), \tag{5}$$

$$\eta_n(r) = \left[\left(\frac{2\alpha_n}{\xi_V}r\right)^{-1} + \left(\frac{\pi}{2} - |\varepsilon_n|\right)^{-1}\right]^{-1}, \tag{6}$$

$$\lambda_n(r) = \frac{1}{2}\left(1 - \frac{\varepsilon_n^2}{2}\right)\left[\frac{2}{\xi_V}r - \frac{1}{\beta_n}\tanh\left(\frac{2\beta_n}{\xi_V}r\right)\right]. \tag{7}$$

α_n and β_n are

$$\alpha_n = -\frac{\pi}{4} + \sqrt{\frac{1}{2}\frac{1-\mu_n^3}{1-\mu_n^2}}, \tag{8}$$

$$\beta_n = \sqrt{\frac{\alpha_n/2}{1-\varepsilon_n^2/2}}, \tag{9}$$

where $\mu_n = |E_F\sqrt{2}/((n+1/2)\Delta_0)|$. The level spacing $\Delta_0^2/(2E_F)$ in Eq. (2) is in agreement with the result $\approx 0.85\Delta_0^2/(2E_F)$ from Eq. (10) in Ref. [4] with $\Delta(\mathbf{r}) = \Delta_0\tanh(r/\xi_V)e^{-i\theta}$. We set $\Delta_0 = 1$ meV, $\xi_V = 30$ nm, and $k_F = 9$ nm^{-1}.

The BdG equation for the Fermi level lying in an elec-

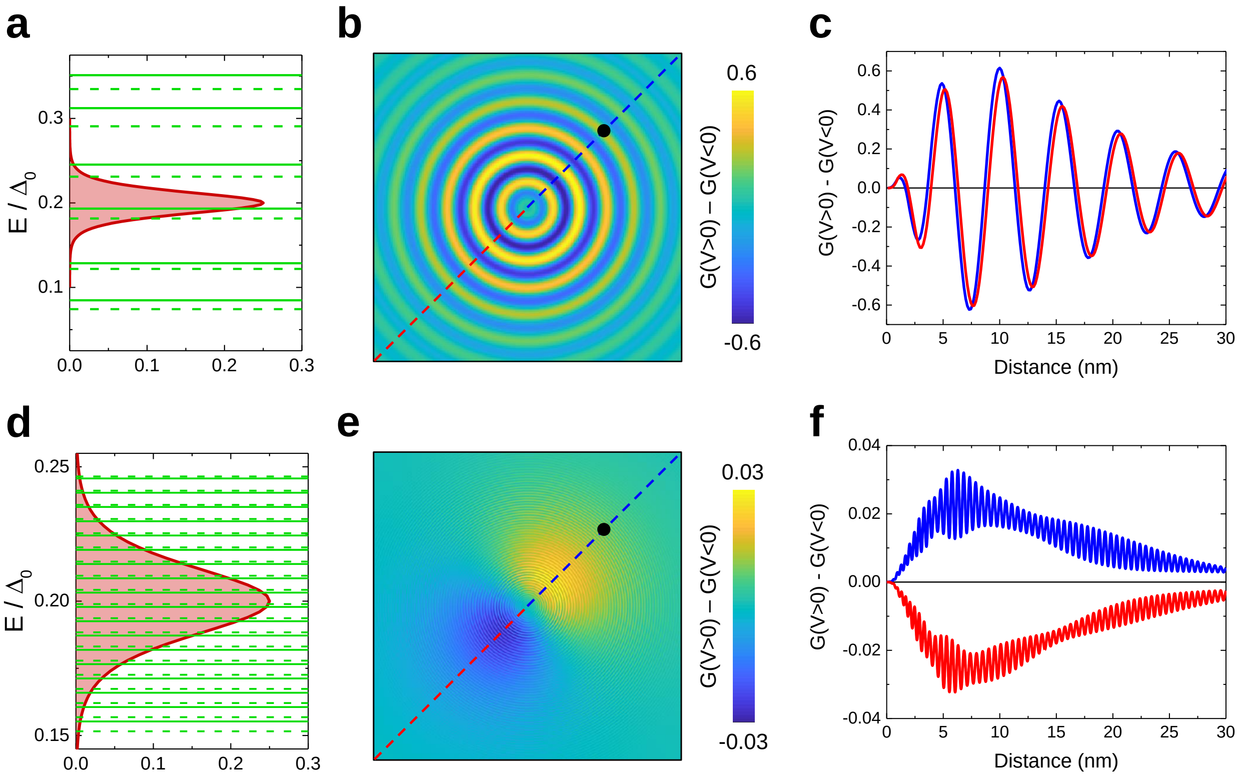

Supplementary Figure 1. **Quantum limit vs thermal broadening.** **a** Vortex bound states are shown as green lines (spin up states as solid lines and spin down states as dashed lines). The derivative of the Fermi function is shown in red. We consider a Fermi wavelength $\lambda_F = 10$ nm, a temperature of 100 mK and a vortex size ξ_V=30 nm. The resulting difference between the tunneling density of states at positive and negative bias voltages (at V=±0.2mV) is shown in **b**, with an impurity located at 20 nm from the vortex center (black dot). The color scale is shown by the bar at the right. We observe the oscillatory electron-hole asymmetry that is characteristic of vortex bound states. In **c** we trace in blue the difference between electron and hole states along a line from the vortex center towards the impurity (blue dashed line in **b**) and in red along the opposite direction (red dashed line in **b**). In **d-e** we show the same results, with the same parameters, except that we take $\lambda_F = 1$nm.

tron like band in the basis $\phi = (\phi^+, \phi^-)^T$ is

$$\begin{pmatrix} \frac{\hbar^2 \mathbf{k}^2}{2m^*} - E_F & \Delta(\mathbf{r}) \\ \Delta(\mathbf{r})^* & -\frac{\hbar^2 \mathbf{k}^2}{2m^*} + E_F \end{pmatrix} \begin{pmatrix} \phi^+(\mathbf{r}) \\ \phi^-(\mathbf{r}) \end{pmatrix} = E \begin{pmatrix} \phi^+(\mathbf{r}) \\ \phi^-(\mathbf{r}) \end{pmatrix}. \tag{10}$$

The functions ψ and ϕ are related by a transformation,

$$\begin{pmatrix} \phi^+(\mathbf{r}) \\ \phi^-(\mathbf{r}) \end{pmatrix} = \mathcal{C} \begin{pmatrix} 0 & 1 \\ 1 & 0 \end{pmatrix} \begin{pmatrix} \psi^+(\mathbf{r}) \\ \psi^-(\mathbf{r}) \end{pmatrix} = \begin{pmatrix} \psi^{-*}(\mathbf{r}) \\ \psi^{+*}(\mathbf{r}) \end{pmatrix}, \tag{11}$$

where $\mathcal{C}$ is the complex conjugate operator. We note that the sign of phase shift η_n between ψ^+ and ψ^- along the radial direction shown in Eq. (3) is inverted to $-\eta_n$ for ϕ^+ and ϕ^- when the band character changes from a hole like band to electron like band. We will show below that the difference between the electron and hole components of the LDOS depends on the sign of η_n (see Eq. (19)), leading to the dependence of the axial asymmetry on the band character.

It is useful to remember the consequences of these expressions for the shape of the LDOS at and around a vortex core[4–11]. The electron and hole LDOS follows approximately the sum over all $|\psi_n^+(\mathbf{r})|^2$ and $|\psi_n^-(\mathbf{r})|^2$, respectively, convoluted with the Fermi function (which is shifted from the Fermi level by eV in presence of a bias voltage V). The difference between electron and hole LDOS occurs at the rapid atomic scale oscillation $k_F r$, because of the phase shift induced by η_n. This difference is however washed out in the experiment because $k_B T \gg (E_n - E_{n-1}) \approx \frac{\Delta_0^2}{E_F}$ in 2H-$NbSe_2$, 2H-$NbSe_{1.8}S_{0.2}$ and in many other superconductors. As a result, the LDOS shows a electron-hole symmetric patterns.

In presence of anisotropic pairing, as in 2H-$NbSe_2$, we can take into account the hexagonal symmetry of the crystalline lattice by using [9]

$$\Delta_a(\mathbf{r}) = c_a \Delta_0 \tanh(r/\xi_V) e^{-i\theta} \cos 6\theta. \tag{12}$$

The sixfold symmetry breaks the rotational symmetry of the isotropic pairing $\Delta(\mathbf{r})$ for $V = 0$, as observed in the experiment, but again it leads to axially symmetric solutions.

Including YSR states. We now consider the effect of magnetic impurities. We locate magnetic impurities at $\mathbf{r} = \mathbf{r}_{p_i}$. The impurity Hamiltonian contains a magnetic (J_i) and non-magnetic (K_i) part and we write it as

$$H_{\rm imp} = \sum_i \left(-J_i \hat{s}\cdot\vec{\sigma} + K_i \tau_z\right)\delta(\mathbf{r} - \mathbf{r}_{p_i}), \quad (13)$$

where τ_z is the Pauli matrix in Nambu space. Here the relation between J_i and K_i can be determined by YSR state energy observed in the experiment. Note that the direction of the magnetic moment is specified by a unit vector $\hat{s}$. The eigenvalues and eigenvectors of $\hat{s}\cdot\vec{\sigma}$ are expressed as $\hat{s}\cdot\vec{\sigma}|s\rangle = s|s\rangle$, where $s = \pm 1$ are eigenvalues. For simplicity, we assume that $J_i = J$ and $K_i = K$.

The perturbed energy eigenvalues and eigenstates, $(E_{n,s}, \psi_{n,s}) \to (\tilde{E}_{n,s}, \tilde{\psi}_{n,s})$ can be obtained by solving the following equation constructed in the subspace spanned by the relevant nearest-neighbor states,

$$\begin{pmatrix} E_{n-6} & 0 & W_{n-6,n} & 0 & 0 \\ 0 & E_{n-1} + V^s_{n-1,n-1} & V^s_{n-1,n} & V^s_{n-1,n+1} & 0 \\ W^*_{n-6,n} & V^{s*}_{n-1,n} & E_n + V^s_{n,n} & V^s_{n,n+1} & W_{n,n+6} \\ 0 & V^{s*}_{n-1,n+1} & V^{s*}_{n,n+1} & E_{n+1} + V^s_{n+1,n+1} & 0 \\ 0 & 0 & W^*_{n,n+6} & 0 & E_{n+6} \end{pmatrix} \begin{pmatrix} c_{n-6,s} \\ c_{n-1,s} \\ c_{n,s} \\ c_{n+1,s} \\ c_{n+6,s} \end{pmatrix} = \tilde{E}_{n,s} \begin{pmatrix} c_{n-6,s} \\ c_{n-1,s} \\ c_{n,s} \\ c_{n+1,s} \\ c_{n+6,s} \end{pmatrix}, \quad (14)$$

where

$$V^s_{n,n'} = \int d^2r\ \psi^\dagger_{n,s}(\mathbf{r}) H_{\rm imp} \psi_{n',s}(\mathbf{r}), \quad (15)$$

$$W_{n,n'} = \int d^2r\ \psi^\dagger_{n,s}(\mathbf{r}) \Delta_a(\mathbf{r}) \psi_{n',s}(\mathbf{r}), \quad (16)$$

and the eigenstate has the form

$$\tilde{\psi}_{n,s} = \sum_{j,s} c_{j,s}\psi_{j,s}, \quad (17)$$

with the summation index $j \in \{n-1, n, n+1\}$ for isotropic pairing ($c_a = 0$, 2H-$NbSe_{1.8}S_{0.2}$) and $j \in \{n-6, n-1, n, n+1, n+6\}$ for anisotropic pairing ($c_a \neq 0$, 2H-$NbSe_2$).

The probability density difference between the electron-like ($|\tilde{\psi}^+_{n,s}|^2$) and the hole-like ($|\tilde{\psi}^-_{n,s}|^2$) states is given by

$$\begin{aligned} |\tilde{\psi}^+_{n,s}(\mathbf{r})|^2 - |\tilde{\psi}^-_{n,s}(\mathbf{r})|^2 = \sum_{j,k,s} & c^*_{j,s} c_{k,s} e^{i(j-k)\theta} \\ & \times \left[P_{jk}(r) + Q_{jk}(r)\right], \end{aligned} \quad (18)$$

where $P_{jk}(r)$ and $Q_{jk}(r)$ are $k_F r$-independent and -dependent functions, respectively, given as,

$$\begin{aligned} P_{jk}(r) =& A_j(r)A_k(r)\sin[F_j(r) - F_k(r)] \\ & \times \sin\left[(\eta_j(r) - \eta_k(r))/2\right], \end{aligned} \quad (19)$$

$$\begin{aligned} Q_{jk}(r) =& A_j(r)A_k(r)\sin[2k_F r + F_j(r) + F_k(r)] \\ & \times \sin\left[(\eta_j(r) + \eta_k(r))/2\right]. \end{aligned} \quad (20)$$

Here, we note that $P_{jk}(r)$ and $Q_{jk}(r)$ are responsible for the slowly and rapidly varying parts of the density difference on the scales of ξ_V and $\lambda_F = 2\pi/k_F$, respectively. As we discuss below, we can neglect the rapidly varying part $Q_{jk}(r)$ and concentrate on the slowly varying $P_{jk}(r)$ for the rest of the calculations.

The difference of the normalized conductance between the positive and negative bias voltages is given by

$$\frac{\delta G(\mathbf{r}, V)}{G_0} = \frac{\beta}{2\rho} \sum_{n,s} \left[\tilde{f}_{ns}(V)(|\tilde{\psi}^+_{n,s}(\mathbf{r})|^2 - |\tilde{\psi}^-_{n,s}(\mathbf{r})|^2)\right], \quad (21)$$

where $\beta = 1/(k_B T)$, $\rho = m^*/(\pi\hbar^2)$ is the normal density of states at the Fermi energy, and

$$\tilde{f}_{ns}(V) = \frac{e^{\beta(eV - \tilde{E}_{n,s})}}{\left[1 + e^{\beta(eV - \tilde{E}_{n,s})}\right]^2} - \frac{e^{\beta(eV + \tilde{E}_{n,s})}}{\left[1 + e^{\beta(eV + \tilde{E}_{n,s})}\right]^2}. \quad (22)$$

In the weak perturbation limit, $|c_{n,s}| \gg |c_{n\pm1,s}|$, the density of the perturbed state can be written as

$$\begin{aligned} |\tilde{\psi}^\pm_{n,s}(\mathbf{r})|^2 \approx & |c_{n,s}|^2 |\psi^\pm_{n,s}(\mathbf{r})|^2 \\ & + 2{\rm Re}\left[c^*_{n-1,s} c_{n,s} \psi^{\pm*}_{n-1,s}(\mathbf{r}) \psi^\pm_{n,s}(\mathbf{r})\right] \\ & + 2{\rm Re}\left[c^*_{n,s} c_{n+1,s} \psi^{\pm*}_{n,s}(\mathbf{r}) \psi^\pm_{n+1,s}(\mathbf{r})\right], \end{aligned} \quad (23)$$

where the coefficients are (up to a normalization factor close to one)

$$\begin{aligned} c_{n-1,s} &= \frac{V^{s*}_{n,n+1} V^s_{n-1,n+1} - V^s_{n-1,n}\delta E_{n+1,s}}{D_{n,s}}, \\ c_{n,s} &= 1, \\ c_{n+1,s} &= \frac{V^s_{n-1,n} V^{s*}_{n-1,n+1} - V^{s*}_{n,n+1}\delta E_{n-1,s}}{D_{n,s}}, \end{aligned} \quad (24)$$

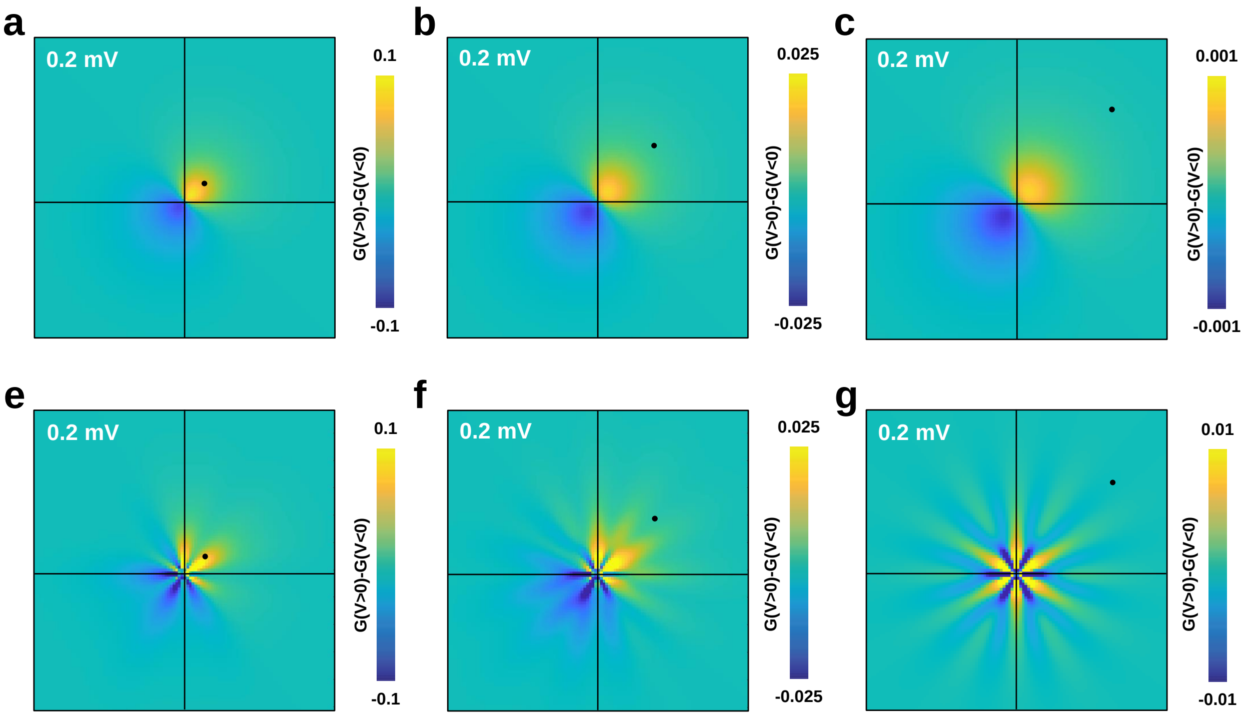

Supplementary Figure 2. **Vortex bound state asymmetry vs. distance of the magnetic impurity from the vortex center.** The calculated difference of the normalized tunneling conductance between positive and negative bias voltages around the vortex center in a field of view of the same size as the ones shown in the main text. The center of the vortex is located at the origin (crossing point between the two black lines) and a single magnetic impurity is marked by a black dot. In **a, e** the impurity is located at 10 nm from the vortex core center and an angle of $\pi/4$, in **b, f** at 30 nm and in **c,g** at 50 nm. Notice the color scale, given by the bars on the right of each figure. The radial asymmetry decreases by several orders of magnitude from **a-c** and from **e-g**.

and

$$\begin{aligned}
\delta E_{n+1,s} =& E_{n+1} + V^s_{n+1,n+1} - \tilde{E}_{n,s} \\
\delta E_{n-1,s} =& E_{n-1} + V^s_{n-1,n-1} - \tilde{E}_{n,s} \\
D_{n,s} =& \delta E_{n+1,s}\, \delta E_{n-1,s} - |V^s_{n-1,n+1}|^2 \\
V^s_{m,n} =& -sJe^{i(m-n)\theta_p} I_{m,n}(r_p) \\
I_{m,n}(r) =& A_m(r) A_n(r) \cos[F_m(r) - F_n(r)] \\
& \times \cos[(\eta_m(r) - \eta_n(r))/2].
\end{aligned} \tag{25}$$

Using Eq. (18), we obtain

$$|\tilde{\psi}^+_{n,s}(\mathbf{r})|^2 - |\tilde{\psi}^-_{n,s}(\mathbf{r})|^2 \approx 4J^2 \cos(\theta - \theta_p) P_{n,n+1}(r) \times \frac{I_{n,n+1}(r_p) I_{n-1,n+1}(r_p)}{D_{n,s}}. \tag{26}$$

In the parameter regime we consider, $F_n(r)$ and $\eta_n(r)$ are monotonically decreasing functions with respect to n and $\eta_n - \eta_{n+1}$ is small and positive of the order of 10^{-2}. In the previous Eq. (25), we ignored the contribution from the non-magnetic potential K in $V^s_{m,n}$ because it contains a small factor $\sin((\eta_m - \eta_n)/2)$. The value of $\cos[F_m(r) - F_n(r)]$ is positive for $0 < F_m(r) - F_{m+1}(r) < \pi/2$ and negative for $\pi/2 < F_m(r) - F_{m+2}(r) < \pi$. The denominator $D_{n,s}$ is in turn negative, so that Eq. (26) leads to positive $P_{n,n+1}(r)$ and $I_{n,n+1}(r_p) I_{n-1,n+1}(r_p)/D_{n,s}$. So we can write

$$|\tilde{\psi}^+_{n,s}(\theta)|^2 - |\tilde{\psi}^-_{n,s}(\theta)|^2 \propto J^2 e^{-ar_p/\xi_V} \cos(\theta - \theta_p), \tag{27}$$

where $a \approx 4(1 - \varepsilon_n^2/2)$. For the case of electron-like bands and within the same simplifying hypothesis one should change $\eta_n(r)$ by $-\eta_n(r)$. It is thus straightforward to conclude that the asymmetry in Eq.(27) would be inverted, i.e. would become $-J^2 e^{-ar_p/\xi_V} \cos(\theta - \theta_p)$.

Let us discuss the implications of these results for the CdGM and YSR states. We show in Supplementary Fig. 1 results at a temperature of 100 mK, with ξ_V =30 nm and $\Delta_0 = 1$ meV. We take a small k_F, or equivalently a large $\lambda_F = 10$ nm in Supplementary Fig. 1**a**-**c**. Then, the thermal broadening is below the CdGM level separation (Supplementary Fig. 1**a**). We see that the resulting plot of the difference $\frac{\delta G(\mathbf{r},V)}{G_0}$ (Supplementary Fig. 1**b**) shows concentring rings, highlighting the electron-hole asymmetry of CdGM states. The rings are

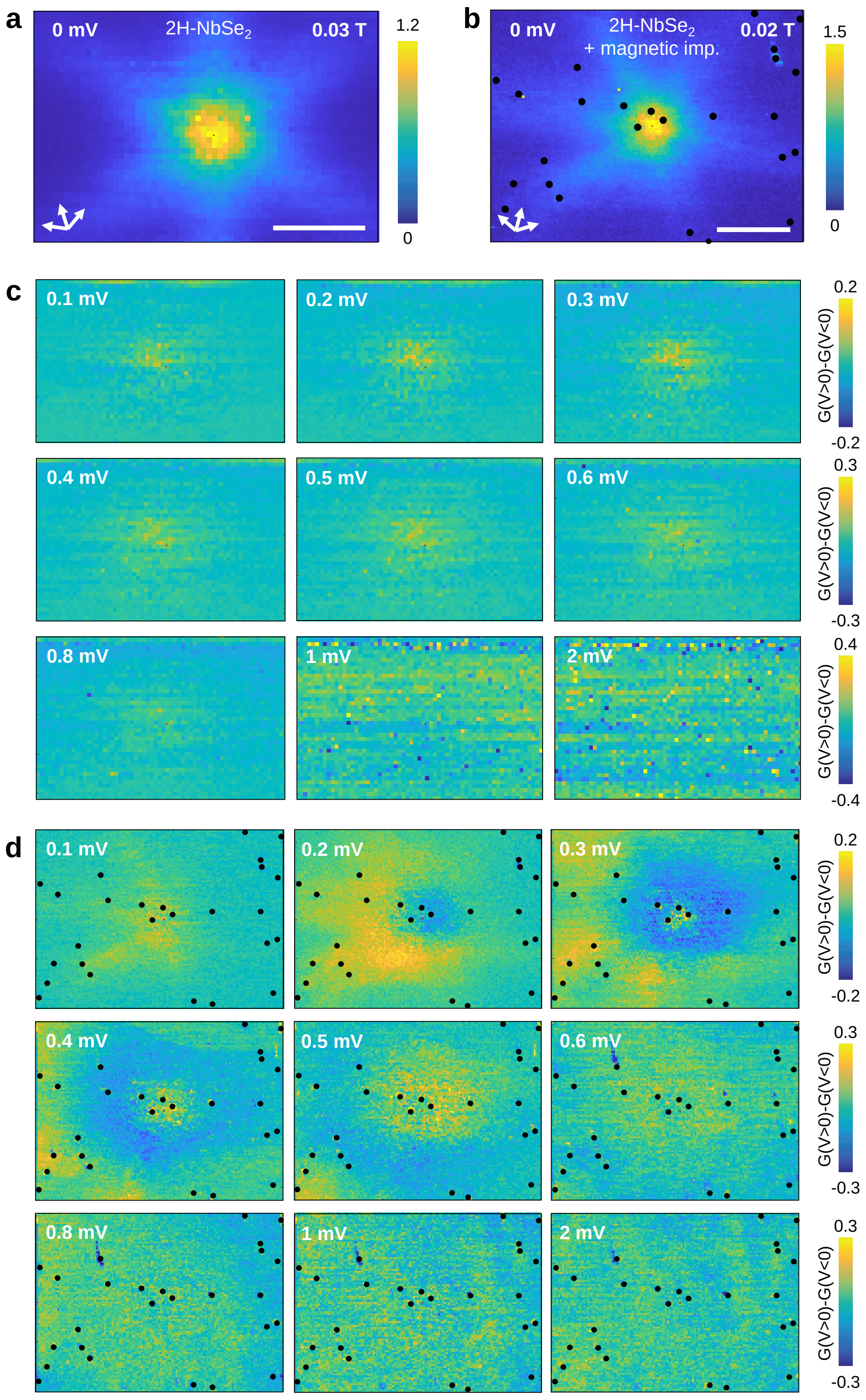

Supplementary Figure 3. **Electron hole symmetry of vortex cores with and without magnetic impurities.** **a** shows a vortex imaged in a field of view without YSR impurities in 2H-NbSe$_2$. We show $\frac{\delta G(\mathbf{r},V)}{G_0}$ of this vortex as a function of the bias voltage (indicated in each panel) in **c**. In **b** we show the same image as in Fig. 4d of the main text and in **d** we provide $\frac{\delta G(\mathbf{r},V)}{G_0}$ as a function of the bias voltage. White scale bars are 20 nm long. The atomic Se lattice directions are shown by three arrows.

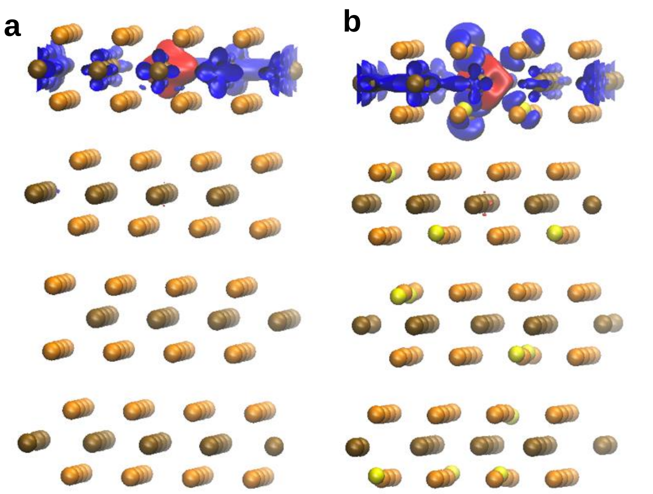

Supplementary Figure 4. **Relaxed atomic positions for 2H-NbSe$_2$ and 2H-NbSe$_{1.8}$S$_{0.2}$ supercells.** We show the atomic structure of the set of slabs used in the calculation, with Nb atoms in ocre, Se in orange and S in yellow, for supercell A **a** and supercell B **b**. Notice that the atomic positions are slightly modified due to the S substitution in **b**. The distribution of S in **b** is random. We also show a lateral view of the spin polarization due to the substituted Fe atom. We plot the spin isosurface corresponding to a spin imbalance in red (spin up) and blue (spin down).

very close to being radially symmetric, with a slight difference (Supplementary Fig. 1**c**) due to the YSR states of the magnetic impurity (black dot in Supplementary Fig. 1). When we change $\lambda_F = 10$ nm to a smaller value, $\lambda_F = 1$ nm (Supplementary Fig. 1**d-f**), the CdGM levels overlap clearly due to thermal broadening (Supplementary Fig. 1**d**). The ring like structure of $\frac{\delta G(\mathbf{r},V)}{G_0}$ nearly vanishes and there is another effect which dominates (Supplementary Fig. 1**e**). This is the radial asymmetry induced by the magnetic impurity and breaks the axial symmetry of the vortex LDOS, with a mirror line that joins the vortex center with the impurity. As we can see in Supplementary Fig. 1**f**, the electron-hole oscillations at λ_F are much weaker and instead the difference conductance $\frac{\delta G(\mathbf{r},V)}{G_0}$ shows a strongly radially asymmetric behavior. Note that the value of $\frac{\delta G(\mathbf{r},V)}{G_0}$ considerably decreases between Supplementary Fig. 1**c** and Supplementary Fig. 1**f**. So that the coupling is particularly well highlighted in presence of thermal broadening.

To understand the influence of the position of the impurity and of gap anisotropy in our result, let us start by considering a single magnetic impurity and isotropic superconducting pairing, $c_a = 0$. We show the result in Supplementary Fig. 2**a-c** for $c_a = 0$. We use $J = -50$ meVnm^2 and $K = 50$ meVnm^2. We see the angular dependence shown in Eq. (27). The impurity induces a stronger electron-hole asymmetry in the CdGM states when it is closer to the center of the vortex. Notice that, when the impurity is far from the vortex core, Supplementary Fig. 2**c**, the corresponding asymmetry decreases very rapidly. Therefore, the impurities closest to the vortex cores determine the axial symmetry breaking. We obtain a qualitatively similar result for an anisotropic superconducting gap ($c_a = 0.05$), Supplementary Fig. 2**d** - **f**, with a coupling that increases when the impurity is located at the vortex center.

To obtain the results shown in the main text, we use ξ_V =30 nm, k_F= 9 nm^{-1}, $E_F = 135$ meV, $\Delta_0 = 1$ meV, $T = 800$ mK for both compounds. Because we usually have many impurities, we introduce their position in the model calculations to add up the effect of each impurity. For 2H-NbSe$_2$, we use $c_a = 0.05$, $J = -10$ meVnm^2 and $K = 10$ meVnm^2, whereas for 2H-NbSe$_{1.8}$S$_{0.2}$ we use $c_a = 0$, $J = -20$ meVnm^2 and $K = 20$ meVnm^2. $\rho = 0.005$ meV^{-1}nm^{-1} is used to fit the experimental data. Notice that the position of impurities is very different in both cases (Fig. 4 of the main text). The different values for J can be associated to difference in the spatial dependence of the wavefunction, which becomes more important when the impurity is close to the vortex center. The actual values of K are not relevant in the calculation of $\frac{\delta G(\mathbf{r},V)}{G_0}$, as we show below.

SUPPLEMENTARY NOTE 2. STM RESULTS WITH AND WITHOUT YSR IMPURITIES.

In Supplementary Fig. 3 we compare the results obtained in a field of view without YSR impurities (Supplementary Fig. 3**a**, **c**), with results with YSR impurities (Supplementary Fig. 3**b**, **d**). We observe that the vortex is, for all $\frac{\delta G(\mathbf{r},V)}{G_0}$, axially symmetric in absence of YSR impurities (Supplementary Fig. 3c) and axially asymmetric (Supplementary Fig. 3**d**) in presence of YSR impurities.

Notice the small electron-hole asymmetry in absence of YSR impurities (Supplementary Fig. 3**a**, **c**), which we discuss below (Supplementary Fig. 11).

SUPPLEMENTARY NOTE 3. COMPUTATIONAL DETAILS OF THE SPIN-POLARIZED ELECTRONIC BANDSTRUCTURE CALCULATIONS

Calculations. We performed spin-polarized first principles calculations based on DFT with the generalized gradient approximation (GGA) of Perdew-Burke-Ernzerhof[12] for the exchange-correlation functional. The plane wave basis sets used projector augmented wave (PAW) pseudopotentials[13] and the electronic wave

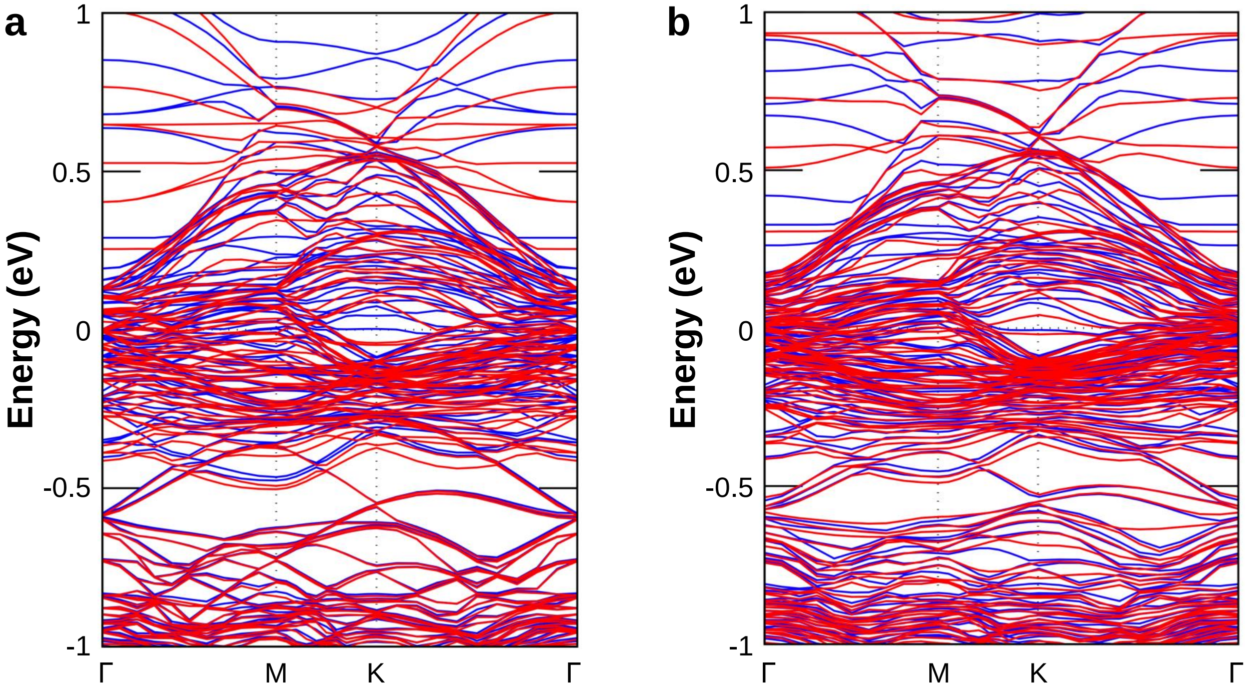

Supplementary Figure 5. **Spin resolved bandstructure of 2H-NbSe$_2$ and of 2H-NbSe$_{1.8}$S$_{0.2}$.** In **a** we show the calculated spin-polarized bandstructure for spin-up (blue) and spin-down (red) states and for 2H-NbSe$_2$ with one Fe atom (supercell A). In **b** we show the same quantities for 2H-NbSe$_{1.8}$S$_{0.2}$ with one Fe atom (supercell B).

functions were expanded with well-converged kinetic energy cutoffs of 75 Ry and 500 Ry for the wavefunctions and charge density, respectively. Dispersion interactions to account for van der Waals interactions between the layers were considered by applying semi-empirical Grimme D2 corrections[14].

Relaxed atomic arrangements. To model the experimental system, we constructed slabs of 4×4×2 size formed by four layers (192 atoms each). The relaxed atomic positions are represented in Supplementary Fig. 4, together with a lateral view of the spin isosurfaces discussed in the main text. All the structures were fully optimized without constraints until the forces on each atom were smaller than 10^3 Ry/au and the energy difference between two consecutive relaxation steps less than 10^4 Ry. The Brillouin zone was sampled by a Γ centered $3 \times 3 \times 1$ k-point Monkhorst-Pack [15] mesh for structural optimization and $6 \times 6 \times 2$ for the self-consistent field (SCF) calculations. We built two supercells. The supercell A is formed by a single Fe impurity substituting a Nb atom and 63 Nb and 128 Se atoms. The supercell B includes a 10% random substitution of Se by S atoms, resulting in 1 Fe, 63 Nb, 115 Se and 13 S atoms.

Magnetic interactions. We inset a vacuum of 18 Å in between sets of cells, to avoid interaction between replica images as a result of periodic boundary conditions. In order to describe the strong correlation of electrons in Mott-Hubbard physics, we adopted a DFT+U approach, where U is the on-site Coulomb repulsion, using the simplified version proposed by Dudarev et al[16]. The Hubbard U was estimated self-consistently using density functional perturbation theory[17]. The distance with the nearest periodic image is 1.4 nm, which is enough to discard any kind of interaction between the Fe ions. Generally, Nb atoms carry only small magnetic moments, that are oppositely oriented to the Fe moments. On the lateral edges of the supercell, however, we observe a polarization of Nb atoms which is small and might be influenced by the neighboring Fe sites. All calculations were carried out in the QuantumEspresso code[18].

Bandstructure. In the Supplementary Fig. 5**a**, **b** we plot the resulting spin resolved bandstructures of 2H-NbSe$_2$ and of 2H-NbSe$_{1.8}$S$_{0.2}$ over the whole supercells. We see that the overall energy dependence of the density of states integrated over the whole Brillouin zone is very similar for both cases. The orbital dependent partial densities of states of orbitals that are close to the Fermi level (Se-3p, Nb-4d and S-3p), show nearly the same values for both spin orientations (Supplementary Fig. 6), although there are slight but visible differences in the Nb and Se partial densities of states, corresponding to the spin polarization of the atoms located close to the Fe impurity. The result on the atomic Fe-3d orbitals shows a clear spin polarization (inset of Supplementary Fig. 6).

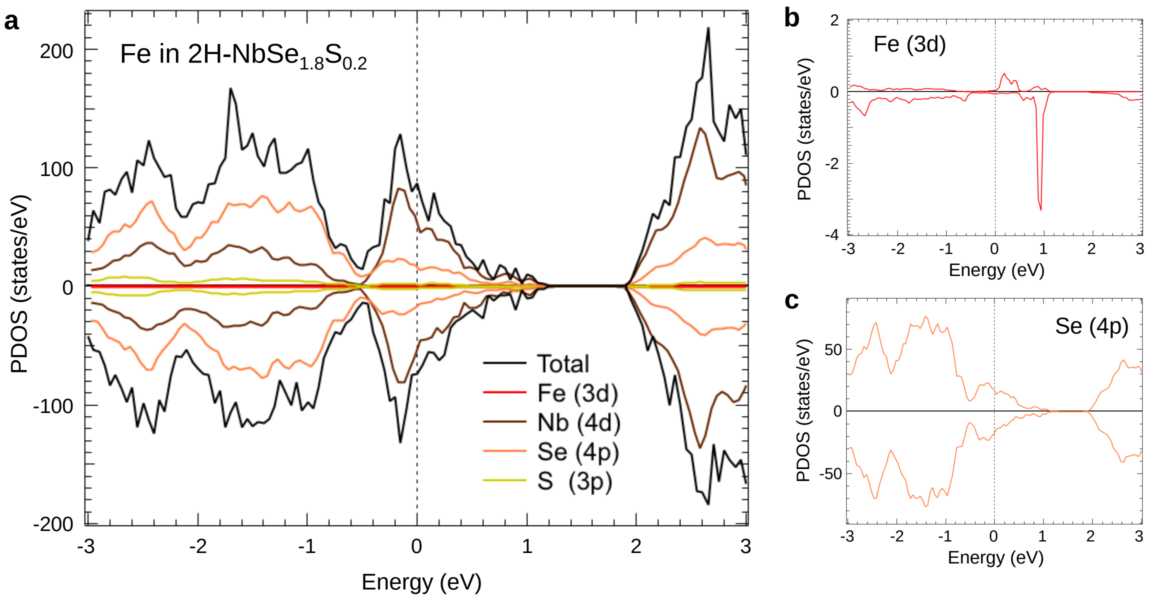

Supplementary Figure 6. **Partial densities of states of atomic species in 2H-NbSe$_{1.8}$S$_{0.2}$.** **a** Spin-polarized partial densities of states integrated over the Brillouin zone for the orbitals marked in the figure for 2H-NbSe$_{1.8}$S$_{0.2}$ with one Fe atom (supercell B). The result for the Fe and Se orbitals is shown in **b** and **c** respectively.

Magnetic moment. In the Supplementary Fig. 7 we show the dependence of the induced magnetic moment with U. We use a reduced system formed by a 4 × 4 slab of monolayer 2H-NbSe$_2$ or of 2H-NbSe$_{1.8}$S$_{0.2}$ containing one Fe impurity that substitutes one Nb. We see that we reach convergence above about 7eV. We use U = 7.5681 eV in our calculations. However, it is also relevant to remark that the magnetic moment remains large already at relatively small values of U.

Calculations on a Fe located on top of a Se atom. We have also explored the situation where the Fe atom does not substitute a Nb atom. For that, we have fully optimized the 4×4×2 slabs of 2H-NbSe$_2$ and 2H-NbSe$_{1.8}$S$_{0.2}$, without a substitutional Fe atom. We have then placed the Fe atom on top of a Se atom in both slab calculations, obtaining an equilibrium position of 2.27 Å above the Se surface. In this scenario, we observe that the spin exchange is much smaller than the one we find when the Fe substitutes a Nb atom. This results in a small spin polarization due to the exchange interaction despite the large magnetic moment ($\approx$2.80 μ_B) localized at the impurity. What is more important is that the shape of the spin density distribution is nearly identical in both cases (Supplementary Fig. 8). This cannot explain the anisotropy in the YSR states observed in Supplementary Fig. 3**c**, **d** of the publication, which are more compatible with Fe substituting a Nb atom.

Atomic size Fe impurity. It is quite useful to see the position of the Fe impurities in the experiment. As we show in Supplementary Fig. 9 we show topographic and tunneling conductance maps with atomic resolution at

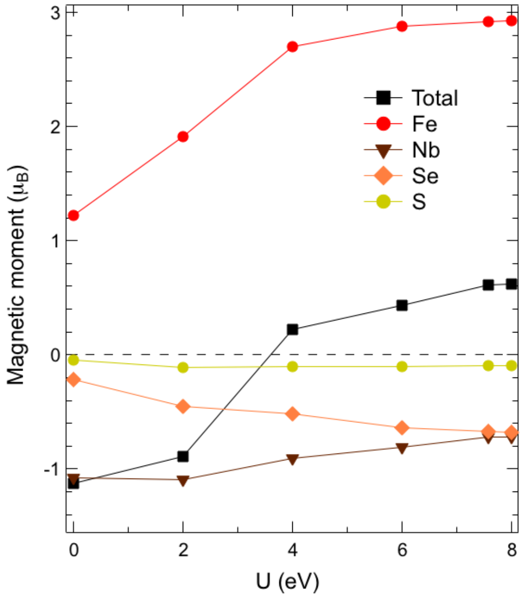

Supplementary Figure 7. **U parameter in calculations.** Magnetic moment as a function of the U parameter for 2H-NbSe$_{1.8}$S$_{0.2}$ with one Fe atom (supercell B).

a Fe impurity (marked by a red dot in Supplementary Fig. 9**b**, **c**). Whereas we can easily identify the Fe impurity from the influence of the YSR on the tunneling conductance, Supplementary Fig. 9**c**, the corresponding topographic STM image, Supplementary Fig. 9**b**, presents very slight or no features, suggesting that the Fe is below

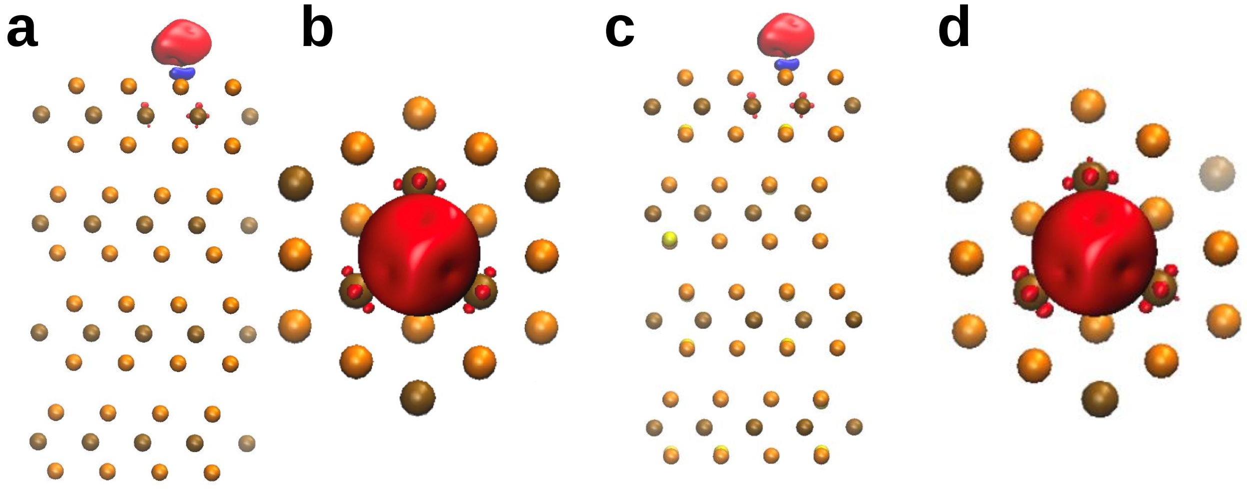

Supplementary Figure 8. **Fe located on top of the Se surface.** Lateral (**a**) view and view from the top (**b**) of the spin density of a Fe atom located on top of the Se surface in 2H-$NbSe_2$ and in 2H-$NbSe_{1.8}S_{0.2}$ (**c**, **d**). Se atoms are shown in orange, Nb atoms in ochre and S atoms in yellow. We plot the spin isosurface corresponding to a spin imbalance by 0.002 in red (spin up) and blue (spin down).

the surface and in between the Se triangles, i.e. at the position of Nb atoms in the layer immediately below the surface Se layer.

SUPPLEMENTARY NOTE 4. STM MEASUREMENTS

To perform the STM measurements we prepared a plate like sample and glued it to our sample holder. We glued a piece of alumina on top of the sample and removed it at 4.2 K by pushing the piece with a beam. To this end, we used the movable sample holder described in Ref.[19]. We measured the freshly exposed surface in a cryogenic system with a base temperature of 800 mK. The design of the STM microscope is very similar to the one described in Ref.[19, 20]. We usually work with tunneling conductances of order of 0.1 μS or below. We provide the tunneling conductance normalized to its value well above the gap edge, usually between 4 mV and 10 mV. Magnetic fields are applied perpendicular to the plate-like sample.

SUPPLEMENTARY NOTE 5. CRYSTAL SYNTHESIS AND CHARACTERIZATION

Synthesis. To synthesize samples of the 2H-$NbSe_2$ and of 2H-$NbSe_{1.8}S_{0.2}$ we first mixed powders of Nb, Se (99.999% Se from Alfa-Aesar) and S (99.98% S from Sigma-Aldrich) in a stoichiometric ratio, and sealed these in an evacuated quartz ampoule. We heated from room temperature to 900 °C at 1.5 °C/min. Then, the temperature was kept constant for ten days and the furnace was switched off for cooling. We mixed 4 mmol of the previously synthesized material with iodine as transport agent (iodine concentration of 5 mg/cm^3). We sealed the mixture in an evacuated quartz ampoule and placed it inside a three-zone furnace with the compound in the leftmost zone. The other two zones were heated up in 3 h from room temperature to 800 °C and kept at this temperature for two days. After that we established a gradient of 800 °C / 750° / 775 °C in the three-zone furnace. The temperatures were kept constant for 15 days and the furnace then cooled down naturally.

Characterization. We obtained large single crystals, with lateral sizes in the order of several millimeters. The crystals were analyzed by powder X-ray diffraction and inductively coupled plasma (ICP) mass spectrometry. The experimental powder patterns were refined with the structure of pure 2H-$NbSe_2$ (ICSD 51589) in both 2H-$NbSe_2$ and of 2H-$NbSe_{1.8}S_{0.2}$. We obtained a variation in lattice constants with S doping compatible with literature ($a = 3.4451(3)$Å, $c = 12.542(1)$Å in 2H-$NbSe_2$ and $a = 3.4327(5)$, $c = 12.506(2)$ in 2H-$NbSe_{1.8}S_{0.2}$) [21, 22]. Both a and c parameters of the hexagonal structure decrease by the same ratio, a result that remains when increasing the S concentration[23]. To ascertain that S doping does not introduce defects other than substution, we have estimated the stacking fault density along the c-axis from the X-ray data. Being a van der Waals compound with little coupling between hexagonal 2H-$NbSe_{2-x}S_x$ planes, we can expect most defects to occur along the c-axis. Following Ref. [24], we can distinguish between deformation and growth faults, with respectively proba-

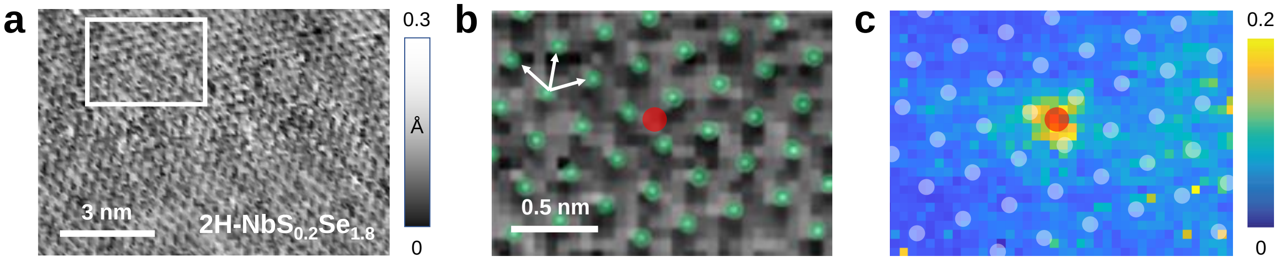

Supplementary Figure 9. **Atomic size map around a Few impurity in 2H-NbSe$_{1.8}$S$_{0.2}$.** In **a** we show a topographic image taken in 2H-NbSe$_{1.8}$S$_{0.2}$ with a bias voltage of 5 mV and a tunneling current of 0.1 nA. In **b** we show a close up view of the area marked by a white rectangle in **a**. The Se atomic lattice is marked by green dots. Atomic directions are marked by white arrows. We mark the position of the Fe impurity by a red dot. **c** Tunneling conductance map at a bias voltage of 0.2 mV. Color scale given by the right bar (we plot the tunneling conductance normalized at bias voltages above the superconducting gap). The position of the Fe impurity is marked by a red dot.

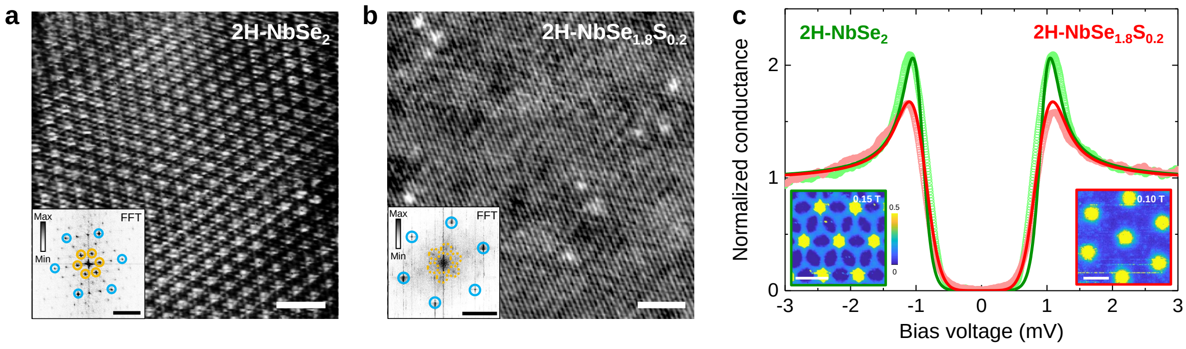

Supplementary Figure 10. **Topography, superconducting gap and vortex lattice in 2H-NbSe$_2$ and 2H-NbSe$_{1.8}$S$_{0.2}$.** **a** Topographic image of pure 2H-NbSe$_2$. In the bottom left inset we show the Fourier transform of the topography. Atomic Bragg spots are marked with blue circles and CDW Bragg spots with orange circles. **b** Similar figure in a field of view of the same size in 2H-NbSe$_{1.8}$S$_{0.2}$. Scales bars on the bottom right are 3 nm long. **c** Normalized tunneling conductance vs bias voltage in 2H-NbSe$_2$ (light green line, T=0.1 K) and in 2H-NbSe$_{1.8}$S$_{0.2}$ (orange line, T=0.8 K). These data are taken at zero field. The zero bias conductance maps showing the vortex lattice under magnetic fields are given in the lower left and right insets, with the color scale given in the lower left inset. Scale bars in the insets are 100 nm long.

bilities μ and ν. Deformation and growth faults can be estimated by analyzing reflections of the type H-K=3N±1. We can then write for the full width at half maximum intensity of the powder scattering Bragg peaks with Miller indices HKL $B_{2\theta}$, $B_{2\theta} = \frac{360}{\pi^2}\tan\theta|L|\left(\frac{d}{c}\right)^2(3\mu+3\nu)$ for even L and $B_{2\theta} = \frac{360}{\pi^2}\tan\theta|L|\left(\frac{d}{c}\right)^2(3\mu+\nu)$ for odd L, with the HKL spacing and $c = 2d_{002}$[24]. We find that the amount of defects along the c-axis is around 2% in both 2H-NbSe$_2$ and 2H-NbSe$_{1.8}$S$_{0.2}$. From inductively coupled plasma (ICP) analysis, we observe 150 ppm of Fe. We do not detect further transition metal impurities within the detection limits of ICP.

The superconducting density of states at zero field of 2H-NbSe$_{1.8}$S$_{0.2}$ shows a smooth distribution of gap values[25]. It is useful to compare the effect of S substitution with the application of pressure in 2H-NbSe$_2$[26, 27]. Pressure increases T_c up to 8.5 K at 10GPa, and then T_c is slightly reduced to 7.5 K at 20 GPa. S substitution by contrast decreases T_c[28]. As shown in Ref. [25], the charge density wave (CDW) of 2H-NbSe$_2$, becomes strongly affected by S substitution in 2H-NbSe$_{1.8}$S$_{0.2}$. In Supplementary Fig. 10**a**, **b** we compare topographic STM images of 2H-NbSe$_2$ with 2H-NbSe$_{1.8}$S$_{0.2}$. 2H-NbSe$_2$ shows CDW order with periodic modulations three times the in-plane lattice constant. In 2H-NbSe$_{1.8}$S$_{0.2}$ we also find CDW order at the same wavevector than for $x = 0$. However, the intensity of the CDW modulation strongly varies with position, producing a disordered CDW pattern. Thus, the S substitution in 2H-NbSe$_{1.8}$S$_{0.2}$ leads to a superconductor which is very similar to 2H-NbSe$_2$,

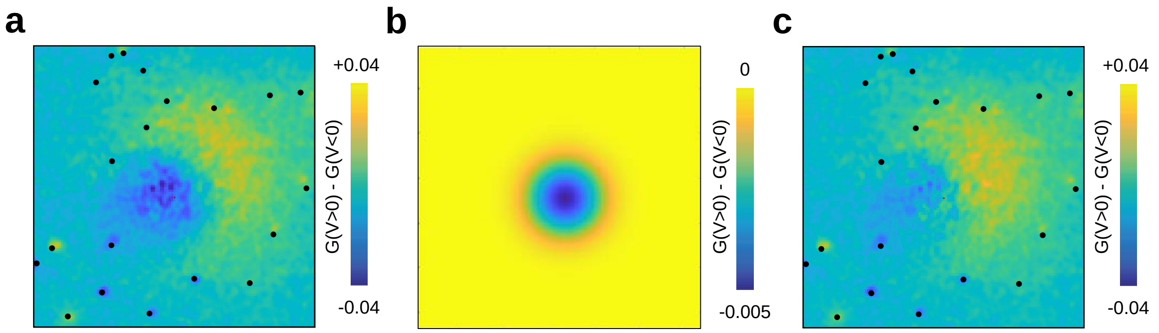

Supplementary Figure 11. **Axially symmetric electron-hole asymmetry.** **a** Difference of the normalized tunneling conductance G between positive and negative bias voltages, $\frac{\delta G(\mathbf{r},V)}{G_0}$ at 0.2 mV is shown as a color scale (bar on the right). **b** Slight electron-hole asymmetric background modelled using a simple gaussian function. **c** Map resulting from the subtraction of **b** from **a**. Black dots in **a** and **c** provide the positions of impurities.

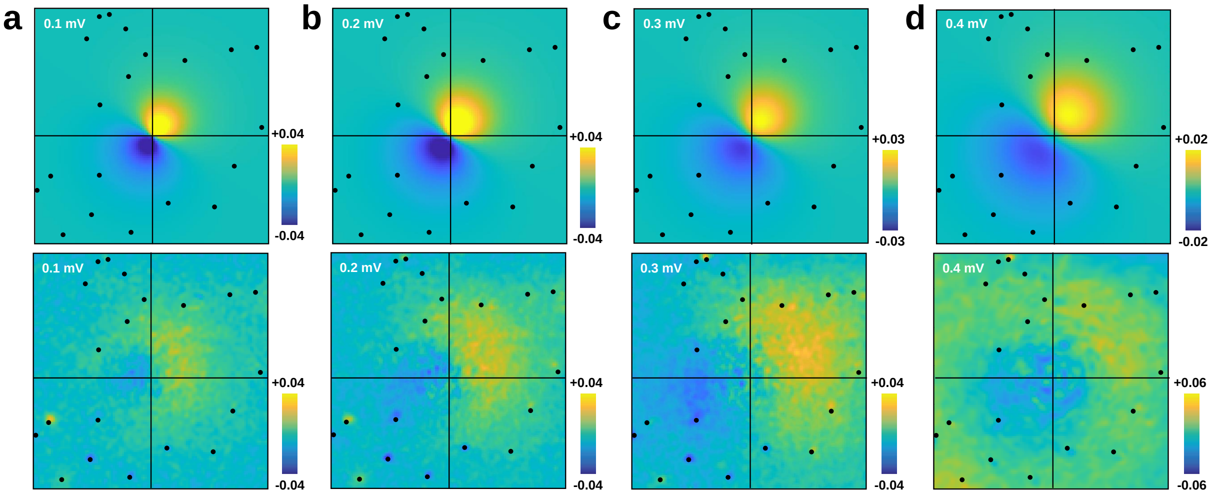

Supplementary Figure 12. **Bias voltage dependence of $\frac{\delta G(\mathbf{r},V)}{G_0}$ in 2H-NbSe$_{1.8}$S$_{0.2}$, compared to our calculations.** We show $\frac{\delta G(\mathbf{r},V)}{G_0} = \frac{G(\mathbf{r},V)-G(\mathbf{r},-V)}{G_0}$ for the bias voltages marked in each panel, obtained from calculations (upper panels) and experiment (lower panels). Black dots provide the position of magnetic impurities. Color scale is given by the bars on the right.

but in-plane isotropic. The mean free path estimated from the residual resistivity is of about $\ell \approx 20$ nm in 2H-NbSe$_{1.8}$S$_{0.2}$, significantly below $\ell \approx 120$ nm in 2H-NbSe$_2$. However, CdGM bound states are well identified in the LDOS[25].

Superconducting gap and vortex lattice. In Supplementary Fig. 10**c** we show the superconducting gap and vortex lattice in pure 2H-NbSe$_2$ and in 2H-NbSe$_{1.8}$S$_{0.2}$. The results in 2H-NbSe$_2$ have been obtained repeatedly in the past (see e.g. [29–31]) and correspond to a superconductor having different values of the gap over the Fermi surface. This is somewhat different in 2H-NbSe$_{1.8}$S$_{0.2}$, which shows a more homogeneous gap distribution. The vortex lattice of 2H-NbSe$_{1.8}$S$_{0.2}$ loses the sixfold star shape characteristic of 2H-NbSe$_2$ and vortices have instead a round shape (bottom insets of Supplementary Fig. 10**c**)[25].

SUPPLEMENTARY NOTE 6. COMPLETE BIAS VOLTAGE DEPENDENCE AND ASYMMETRY IN CLOSE LYING VORTICES.

We have subtracted a radially symmetric signal to $\frac{\delta G(\mathbf{r},V)}{G_0}$ to obtain the images shown in the main text for 2H-NbSe$_{1.8}$S$_{0.2}$. As we show in Supplementary Fig. 11, the radially symmetric electron hole anisotropy in $\frac{\delta G(\mathbf{r},V)}{G_0}$ is very small, of less than 10% of $\frac{\delta G(\mathbf{r},V)}{G_0}$.

For completeness, we provide the results for all bias voltages in Supplementary Fig. 12 (2H-NbSe$_{1.8}$S$_{0.2}$) and in Supplementary Fig. 13 (2H-NbSe$_2$), comparing each time with calculations. We observe that asymmetry

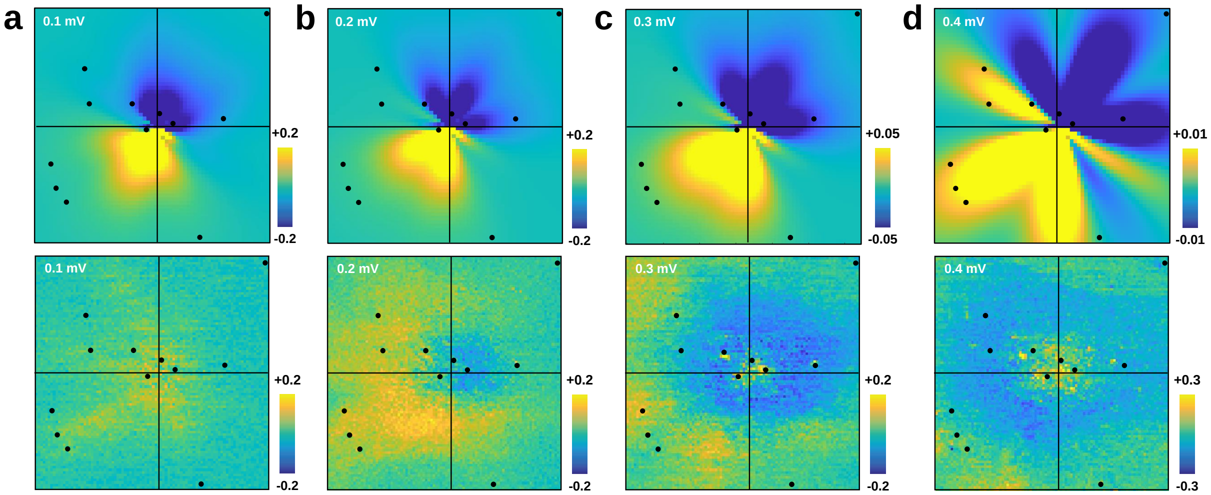

Supplementary Figure 13. **Bias voltage dependence of $\frac{\delta G(\mathbf{r},V)}{G_0}$ in 2H-NbSe$_2$, compared to our calculations**. We show $\frac{\delta G(\mathbf{r},V)}{G_0} = \frac{G(\mathbf{r},V)-G(\mathbf{r},-V)}{G_0}$ for the bias voltages marked in each panel, obtained from calculations (upper panels) and experiment (lower panels). Black dots provide the position of magnetic impurities. Color scale is given by the bars on the right.

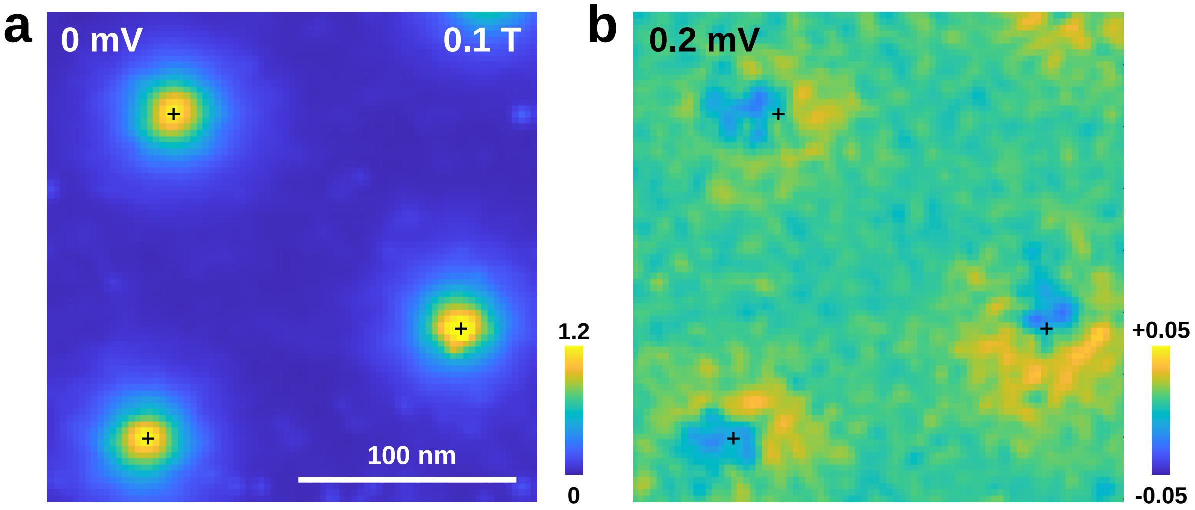

Supplementary Figure 14. **Asymmetry of close lying vortices**. **a** We show three vortices lying close together at a magnetic field of 0.1 T. Vortex cores are marked by black crosses. YSR states from impurities are distributed all over the scanning window. **b** $\frac{\delta G(\mathbf{r},V)}{G_0}$ in the same field of view, with vortex centers marked by black crosses.

spreads to larger distances with increasing bias voltages and that its overall shape is well captured by calculations.

We also provide in Supplementary Fig. 14 results on three close lying vortices in 2H-NbSe$_{1.8}$S$_{0.2}$. Notice that the field of view is much larger than in other images. We see that the asymmetric shape in $\frac{\delta G(\mathbf{r},V)}{G_0}$ extends along different directions (Supplementary Fig. 14**b**) in each vortex, due to a different distribution of magnetic impurities close to the center of each vortex.

SUPPLEMENTARY NOTE 7. MAGNETIC SUSCEPTIBILITY MEASUREMENTS AND LARGE SIZE CONDUCTANCE MAPS AT ZERO FIELD AND UNDER MAGNETIC FIELDS IN THE NORMAL PHASE

Magnetic susceptibility of the bulk. We have performed susceptibility measurements in the same samples measured by STM using a Quantum Design PPMS system, with the magnetic field applied perpendicular to the plate like sample. Inside the superconducting phase, the signal is dominated by the superconducting diamagnetic response. Above T_c we observe a diamagnetic background and a small signal which is ferromagnetic like (Supplementary Fig. 15**a**). We can extract this small ferromagnetic like component from the background and compare its size with the expected Fe moment, taking a Fe concentration of 150 ppm. It is quite remarkable that, although being clearly a very rough approximation which might be strongly influenced by clustering at edges or on large defects induced during growth, we obtain a saturation magnetization with a moment of about a Bohr magneton per Fe ion, compatible with the value found in the theoretical calculations (Supplementary Fig. 7). Previous measurements with much larger (30%) Fe concentration report values up to five Bohr magnetons[32], which are compatible with a Fe^{4+} valence. The reduced magnetic moment obtained here points to a reduction of the Fe valence, which might be chemically compensated by Se vacancies. More recently, the substitional exchange of Fe atoms in transition metal dichalcogenide MoS_2 has been studied in detail, observing directly the exchange of transition metal atoms and finding similarly spin-polarized electronic bandstructure in small cells of transition metal dichalcogenide layers containing one Fe atom[33].

Maps at zero field and in the normal phase. We provide a large size zero bias conductance map at zero field (Supplementary Fig. 15**b**) and under magnetic fields above the critical field of 2H-$NbSe_{1.8}S_{0.2}$ (Supplementary Fig. 15**c**). These show that impurities are generally well separated. Furthermore, impurities do not influence the zero bias density of states of the normal phase above the critical field.

[1] Johannes, M. D., Mazin, I. I. & Howells, C. A. Fermi-surface nesting and the origin of the charge-density wave in $NbSe_2$. *Phys. Rev. B* **73**, 205102 (2006).

[2] Fletcher, J. D. *et al.* Penetration depth study of superconducting gap structure of $2H{-}NbSe_2$. *Phys. Rev. Lett.* **98**, 057003 (2007).

[3] Majumdar, A. *et al.* Interplay of charge density wave and multiband superconductivity in layered quasi-two-dimensional materials: The case of $2H{-}NbS_2$ and $2H{-}NbSe_2$. *Phys. Rev. Materials* **4**, 084005 (2020).

[4] Caroli, C., deGennes, P. G. & Matricon, J. Bound fermion states on a vortex line in a type II superconductor. *Physics Letters* **9**, 307 – 309 (1964).

[5] Bardeen, J., Kümmel, R., Jacobs, A. E. & Tewordt, L. Structure of vortex lines in pure superconductors. *Phys. Rev.* **187**, 556–569 (1969).

[6] Clinton, W. L. Approximate solutions for the Bogoliubov de Gennes equations: Superconductor normal metal superconductor junctions and the vortex problem. *Phys. Rev. B* **46**, 5742–5745 (1992).

[7] Gygi, F. & Schlüter, M. Self-consistent electronic structure of a vortex line in a type-II superconductor. *Phys. Rev. B* **43**, 7609–7621 (1991).

[8] Fischer, Ø., Kugler, M., Maggio-Aprile, I., Berthod, C. & Renner, C. Scanning tunneling spectroscopy of high-temperature superconductors. *Rev. Mod. Phys.* **79**, 353–419 (2007).

[9] Hayashi, N., Isoshima, T., Ichioka, M. & Machida, K. Low-lying quasiparticle excitations around a vortex core in quantum limit. *Phys. Rev. Lett.* **80**, 2921–2924 (1998).

[10] Rainer, D., Sauls, J. A. & Waxman, D. Current carried by bound states of a superconducting vortex. *Phys. Rev. B* **54**, 10094–10106 (1996).

[11] Bespalov, A. A. & Plastovets, V. D. Large spectral gap and impurity-induced states in a two-dimensional Abrikosov vortex. *Phys. Rev. B* **103**, 024510 (2021).

[12] Perdew, J. P., Burke, K. & Ernzerhof, M. Generalized gradient approximation made simple. *Phys. Rev. Lett.* **77**, 3865–3868 (1996).

[13] Kresse, G. & Joubert, D. From ultrasoft pseudopotentials to the projector augmented-wave method. *Phys. Rev. B* **59**, 1758–1775 (1999).

[14] Grimme, S. Semiempirical gga-type density functional constructed with a long-range dispersion correction. *Journal of Computational Chemistry* **27**, 1787–1799 (2006).

[15] Monkhorst, H. J. & Pack, J. D. Special points for Brillouin-zone integrations. *Phys. Rev. B* **13**, 5188–5192 (1976).

[16] Dudarev, S. L., Botton, G. A., Savrasov, S. Y., Humphreys, C. J. & Sutton, A. P. Electron-energy-loss spectra and the structural stability of nickel oxide: An lsda+u study. *Phys. Rev. B* **57**, 1505–1509 (1998).

[17] Timrov, I., Marzari, N. & Cococcioni, M. Hubbard parameters from density-functional perturbation theory. *Phys. Rev. B* **98**, 085127 (2018).

[18] Giannozzi, P. *et al.* QUANTUM ESPRESSO: a modular and open-source software project for quantum simulations of materials. *Journal of Physics: Condensed Matter* **21**, 395502 (2009).

[19] Suderow, H., Guillamón, I. & Vieira, S. Compact very low temperature scanning tunneling microscope with mechanically driven horizontal linear positioning stage. *Review of Scientific Instruments* **82** (2011).

[20] Galvis, J. A. *et al.* Three axis vector magnet set-up for cryogenic scanning probe microscopy. *Review of Scientific Instruments* **86** (2015).

[21] Hotje, U. & Binnewies, M. Chemischer transport fester lösungen. 23 [1] der chemische transport von mischphasen im system MoS2/MoSe2, MoS2/NbS2, MoSe2/NbSe2 und NbS2/NbSe2. *Zeitschrift fr anorganische und allgemeine Chemie* **631**, 2467–2474 (2005).

[22] Jones, R. E., Shanks, H. R., Finnemore, D. K. & Morosin, B. Pressure effect on superconducting $nbse_2$ and nbs_2.

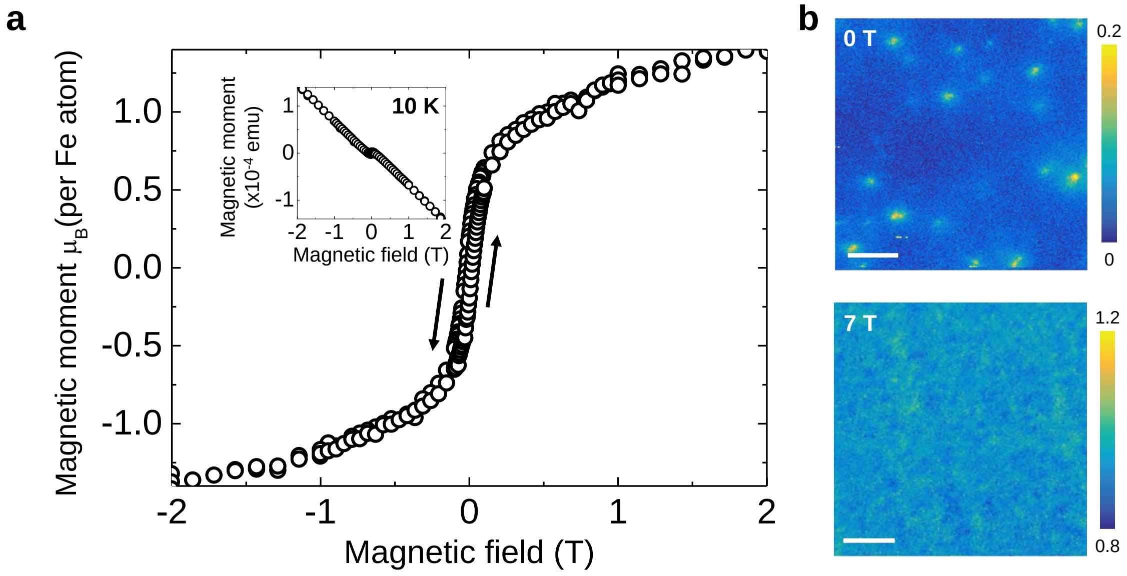

Supplementary Figure 15. **Macroscopic magnetic susceptibility of Fe impurities in 2H-NbSe$_{1.8}$S$_{0.2}$. a** We show as circles the magnetic moment as a function of the magnetic field at 10 K. To obtain this curve, we have subtracted a diamagnetic background from the magnetization as a function of the magnetic field (shown in the inset) and divided by the estimated concentration of Fe atoms in the sample, taking the 150 ppm value determined from inductively coupled plasma analysis. Arrows show the direction of the field sweep. **b** Tunneling conductance at zero bias normalized to the tunneling conductance at large bias at zero field (top panel) and at 7 T (bottom panel). Color scale (bars at the right) show the normalized conductance values.

Phys. Rev. B **6**, 835–838 (1972).

[23] S. Mañas et al., in preparation.

[24] Warren, B. E. *X-ray Diffraction* (Courier Corporation, 1990).

[25] Fente, A. *et al.* Field dependence of the vortex core size probed by scanning tunneling microscopy. *Phys. Rev. B* **94**, 014517 (2016).

[26] Suderow, H., Tissen, V. G., Brison, J. P., Martínez, J. L. & Vieira, S. Pressure induced effects on the Fermi surface of superconducting 2H$-$NbSe$_2$. *Phys. Rev. Lett.* **95**, 117006 (2005).

[27] Moulding, O., Osmond, I., Flicker, F., Muramatsu, T. & Friedemann, S. Absence of superconducting dome at the charge-density-wave quantum phase transition in 2$H-$NbSe$_2$. *Phys. Rev. Research* **2**, 043392 (2020).

[28] Cho, K. *et al.* Using controlled disorder to probe the interplay between charge order and superconductivity in NbSe$_2$. *Nature Communications* **9**, 2796 (2018).

[29] Hess, H. F., Robinson, R. B., Dynes, R. C., Valles, J. M. & Waszczak, J. V. Scanning-tunneling-microscope observation of the Abrikosov flux lattice and the density of states near and inside a fluxoid. *Phys. Rev. Lett.* **62**, 214–216 (1989).

[30] Hess, H. F., Robinson, R. B. & Waszczak, J. V. Vortex-core structure observed with a scanning tunneling microscope. *Phys. Rev. Lett.* **64**, 2711–2714 (1990).

[31] Guillamon, I., Suderow, H., Guinea, F. & Vieira, S. Intrinsic atomic-scale modulations of the superconducting gap of 2H-NbSe$_2$. *Phys. Rev. B* **77**, 134505 (2008).

[32] Voorhoeve-van Den Berg, J. & Sherwood, R. Low-temperature magnetic susceptibilities of NbSe$_2$ containing the first-row transition metals. *Journal of Physics and Chemistry of Solids* **32**, 167 – 173 (1971).

[33] Fu, S. *et al.* Enabling room temperature ferromagnetism in monolayer MoS$_2$ via in situ iron-doping. *Nature Communications* **11**, 2034 (2020).
